# CD28 Superagonistic Activation of T Cells Induces a Tumor Cell-Like Metabolic Program

**DOI:** 10.1089/mab.2018.0042

**Published:** 2019-04-22

**Authors:** Thilipan Thaventhiran, Wai Wong, Ahmad F. Alghanem, Naif Alhumeed, Mohammad A. Aljasir, Simeon Ramsey, Swaminathan Sethu, Han Xian Aw Yeang, Amy E. Chadwick, Michael Cross, Steven D. Webb, Laiche Djouhri, Christina Ball, Richard Stebbings, Jean G. Sathish

**Affiliations:** ^1^ Medical Research Council Centre for Drug Safety Science and Department of Molecular and Clinical Pharmacology, University of Liverpool, Liverpool, United Kingdom.; ^2^ Inflammation and Remodeling, Pfizer Research Unit, Cambridge, Massachusetts.; ^3^ Department of Physiology, College of Medicine, King Saud University, Riyadh, Saudi Arabia.; ^4^ National Institute for Biological Standards and Control, Hertfordshire, United Kingdom.

**Keywords:** CD4^+^ effector memory T cells, CD28 superagonist, lipogenesis, glycolysis, oxidative phosphorylation

## Abstract

CD28 superagonist (CD28SA), a therapeutic immunomodulatory monoclonal antibody triggered rapid and exaggerated activation of CD4^+^ effector memory T cells (T_EMs_) in humans with unwanted serious adverse effects. It is well known that distinct metabolic programs determine the fate and responses of immune cells. In this study, we show that human CD4^+^ T_EMs_ stimulated with CD28SA adopt a metabolic program similar to those of tumor cells with enhanced glucose utilization, lipid biosynthesis, and proliferation in hypoxic conditions. Identification of metabolic profiles underlying hyperactive T cell activation would provide a platform to test safety of immunostimulatory antibodies.

## Introduction

CD28 superagonists (CD28SAs) are monoclonal antibodies (mAbs) that engage CD28 costimulatory receptors resulting in potent T cell activation independent of concomitant T cell receptor (TCR) engagement.^(1)^ TGN1412, a CD28SA, triggered an excessive and adverse cytokine release (cytokine storm) when administered to volunteers in a human Phase 1 clinical trial.^(2)^ We and others have shown that CD28SA induces exaggerated activation, polyclonal expansion, and migration of CD4^+^ effector memory T cells (T_EMs_).^(2–4)^ The basis for this hyperactive, dysregulated phenotype is subject of much research and the lack of inhibitory inputs^(4)^ has been suggested as one potential mechanism. T cells need to undergo metabolic reprogramming to adapt to their changing metabolic needs as they progress from resting T cells to fully differentiated effectors and memory cells.^(5,6)^

T cells utilize glucose as the primary nutrient source for energy generation and biomass production, although amino acids, such as glutamine, are also utilized.^(5)^ Before activation, naive T cells have low metabolic requirements and rely on mitochondrial oxidative pathways for basal energy generation. Upon activation, T cells adopt a metabolic profile typified by aerobic glycolysis and basal oxidative phosphorylation (OXPHOS).^(5,7)^ Rapidly proliferating T cells require lipids to support membrane biogenesis and depending on the T cell subset, lipids may be acquired or synthesized (lipogenesis).^(8)^ T cells use distinct metabolic programs according to their differentiation state and immunological role. Studies have shown that CD4^+^ T helper (Th)1, Th2, and Th17 cells are highly glycolytic, whereas CD4^+^ regulatory T cells have high lipid oxidation rates.^(9)^ The metabolic phenotype of the TGN1412-target cell, CD4^+^ T_EMs_, is yet to be fully characterized. Importantly, the metabolic program that supports the dramatic hyperactivation and proliferation induced by CD28SA is currently unknown.

Tumor cells exhibit metabolic abnormalities such as elevated aerobic glycolysis and *de novo* fatty acid biosynthesis to generate the energy required to support rapid cell division.^(10)^ In this report, we demonstrate that superagonistic activation programs CD4^+^ T_EMs_ toward a tumor cell-like metabolic profile that favors enhanced glycolysis and lipogenesis. We also define ATP-citrate lyase (ACL) and acetyl-Coenzyme A (ACC) as key molecular indicators of the CD28SA-induced lipogenic phenotype.

## Materials and Methods

### Reagents

All reagents were obtained from Sigma-Aldrich (United Kingdom) unless otherwise stated.

### Effector memory T cell isolation

Ethics approval for the use of human peripheral blood mononuclear cells (PBMCs) from healthy donors was given by the local Ethics Committee and all subjects provided informed consent. PBMCs were isolated from heparinized venous blood by density gradient separation (LymphoPrep, O7811; Axis-Shield). The CD4^+^ T_EMs_ isolation kit (130–094–125; Miltenyi Biotec) was used to purify T_EMs_ from PBMCs according to the manufacturer's instructions.

### Stimulating antibodies

Humanized superagonistic anti-CD28 antibody, NIB1412, a human IgG4 sharing the H chain V region and L chain sequences of TGN1412, was generated at the National Institute for Biological Standards and Control (NIBSC, United Kingdom). Murine anti-human CD3 (clone: UCHT1, Cat No. 16–0038–85) antibody was purchased from eBioscience (United Kingdom).

### Proliferation assays

Plate-bound or solid-phase PBMC *in vitro* systems have been previously shown to support robust T cell activation by anti-CD3 and CD28SA,^(4,11)^ and therefore this system was chosen to study metabolic reprogramming of T_EM_ cells. Ninety-six-well round-bottom non tissue culture treated plates were coated with stimulating antibodies at 37°C for 2 hours. Plates were washed twice to remove unbound antibody before addition of T cells. The T cells were cultured in complete media (RPMI 1640 supplemented with 15% fetal calf serum (Life Technologies, United Kingdom), 2 mM l-glutamine, 50 U/mL penicillin, and 0.05 mg/mL streptomycin) for 72 hours (at 37°C) in either normoxic (20% O_2_) or hypoxic (5% O_2_) conditions. The cells were pulsed with tritiated thymidine ([^3^H]-TdR, 0.5 μCi/well), 18 hours before the end of the indicated time point. Incorporation of [^3^H]-TdR in T cells was determined using a β-scintillation counter (MicroBetaTrilux; PerkinElmer Life Sciences, United Kingdom). Data obtained are represented as mean counts per minute.

### Cell viability assay

Briefly, CD4^+^ T_EMs_ were plated in base media with l-glutamine ± glucose at a density of 5 × 10^4^ cells per well in 96-well plates precoated with anti-CD3 mAbs or NIB1412. Following overnight incubation at 37°C, each sample was collected and assayed for cell viability using Trypan Blue exclusion. Percentage viability was determined by the Countess™ automated cell counter.

### Flow cytometric analysis

CD4^+^ T_EMs_ were activated with plate-bound anti-CD3 or NIB1412 for 48 hours. For the quantification of mitochondria, cells were stained with MitoTracker^®^ Deep Red FM (M22426; Molecular Probes) at 20 nM during the last 30 minutes of treatment. Cells were washed with phosphate-buffered saline (PBS) and fixed with 4% paraformaldehyde and stained with HCS LipidTOX™ Green Neutral Lipid Stain (H34475; Invitrogen) at 1:500. To quantify mitochondrial superoxide production, cells were incubated with MitoSOX™ Red (Cat No. M36008; Invitrogen) at 37°C for 10 minutes, washed and fixed in 2% paraformaldehyde. MitoSOX Red was excited at 488 nm and fluorescence emission at 575 nm was measured.

For the determination of glucose uptake and cell surface expression of glucose transporters, cells were incubated with 2-NBDG (N13195; Molecular Probes) for 30 minutes, washed three times, and then stained with anti-Glut1-PE (MAB1418; R&D Systems) for 20 minutes. Cells were then washed and fixed with 4% paraformaldehyde.

Staining and incubations were performed at 37°C. Untreated cells were used as controls. Fluorescent signals from cells were acquired on BD FACS Canto II flow cytometer and data were analyzed using Cyflogic software v. 1.2.1.

### Immunofluorescence microscopy

Imaging of mitochondria and lipid droplets was performed by washing preactivated cells after 48 hours and plating them on poly-d-lysine (Sigma)-coated cover slips, and stained with MitoTracker Deep Red FM (1:1000) and HCS LipidTOX Green Neutral Lipid Stain (1:2000), respectively. After staining, cells were washed once with PBS, followed by a 10-minute incubation in PBS; this was then replaced with fresh PBS. Fixed cells were also stained with Alexa 568-conjugated anti-GAPDH antibody (D16H11; CST). Cover slips were mounted with Duolink^®^ In Situ Mounting Medium with DAPI (DUO82040; Sigma). Cells in five randomly selected optical fields per replicate were visualized and images were acquired using a Axio Observer Zeiss microscope with objective LD “Plan-Neofluar” 20 × /0.4 Corr Ph2 M27, and analyzed with ZEN Pro 2012 software.

### Gel electrophoresis and western immunoblotting

Cells were lysed with RIPA buffer with 1 mM PMSF and protease inhibitor cocktail. Twenty micrograms of protein lysate was resolved by 10%–12% SDS-PAGE (sodium dodecyl sulfate–polyacrylamide gel electrophoresis), transferred to PVDF membranes (Bio-Rad), blocked, and probed with the primary antibodies: anti-ACL (phospho S455) (#4331; CST) and anti-Acetyl Coenzyme A Carboxylase (phospho S79) (ab68191; Abcam, United Kingdom) followed by appropriate horseradish peroxidase-conjugated secondary antibodies (Cell Signaling Technology, United Kingdom) and visualized using the ECL system (PerkinElmer Life Sciences).

### Bioenergetics of T cells

CD4^+^ T_EMs_ were stimulated with plate-bound anti-CD3 mAb or NIB1412 for 48 hours in complete media and then resuspended in serum-free base media (102353-100; Seahorse Bioscience) with 2 mM l-glutamine ±1 mM sodium pyruvate ±25 mM glucose. Subsequently, cells were seeded in precoated poly-d-lysine (50 μg/mL) XF 96-well microplates (Seahorse Bioscience) at 3 × 10^5^ cells per well, spun down, and inoculated at 37°C for 30 minutes. Bioenergetics of CD4^+^ T_EMs_ were determined using the XF Cell Mito Stress Test Kit (103015-100; Seahorse Bioscience), XF Glycolysis Stress Test Kit (102194-100; Seahorse Bioscience), and a XF^e^96 Analyzer (Seahorse Bioscience). Briefly, following the measurement of basal respiration, Oligomycin was injected to inhibit ATP-synthase to measure the oxygen consumed for ATP production during mitochondrial respiration-coupled OXPHOS of ADP. The addition of the uncoupling agent 2,4-DNP enabled measurement of the maximal respiratory capacity of the cells. The final injection of rotenone and antimycin A blocked the electron transport chain thus revealing nonmitochondrial oxygen consumption. Glycolysis and the glycolytic reserve of cells were measured by the sequential addition of glucose, Oligomycin, and 2-d-glucose. The extracellular acidification rate (ECAR) after glucose injection is the basal rate of glycolysis and was higher in CD28SA-stimulated cells than anti-CD3-stimulated cells. The addition of Oligomycin inhibits mitochondrial ATP production and shifts energy production predominantly to the glycolytic pathway, revealing the maximum glycolytic capacity of the cells. The glycolytic reserve is calculated from the difference between the glycolytic capacity and glycolytic rate.

### Acetyl-Coenzyme A measurement

Concentration of acetyl-CoA in whole-cell lysates from preactivated CD4^+^ T_EMs_ was measured by fluorescence assay using a kit according to the manufacturer's instructions (The PicoProbe Acetyl CoA Assay Kit: ab87546; Abcam). Fluorescence was quantified using a Varioskan™ Flash Multimode Plate Reader (Thermo Scientific, United Kingdom).

### Citrate and alpha-ketoglutarate measurement

Concentration of citrate and alpha-ketoglutarate (α-KG) in whole-cell lysates from preactivated CD4^+^ T_EMs_ was measured by fluorescence assay using a kit according to the manufacturer's instructions (The Citrate Assay Kit: ab83396, α-KG Assay Kit: ab83431; Abcam). Relative fluorescence units/intensity were quantified using a Varioskan Flash Multimode Plate Reader (Thermo Scientific).

### Statistical analysis

Unpaired, two-tailed Student's *t*-test was used to analyze data and results were presented as mean ± standard deviation. *p* < 0.05 was considered to be statistically significant.

## Results

### CD28SA-activated T_EMs_ cells display a hyperactive phenotype

**FIG. 1.**
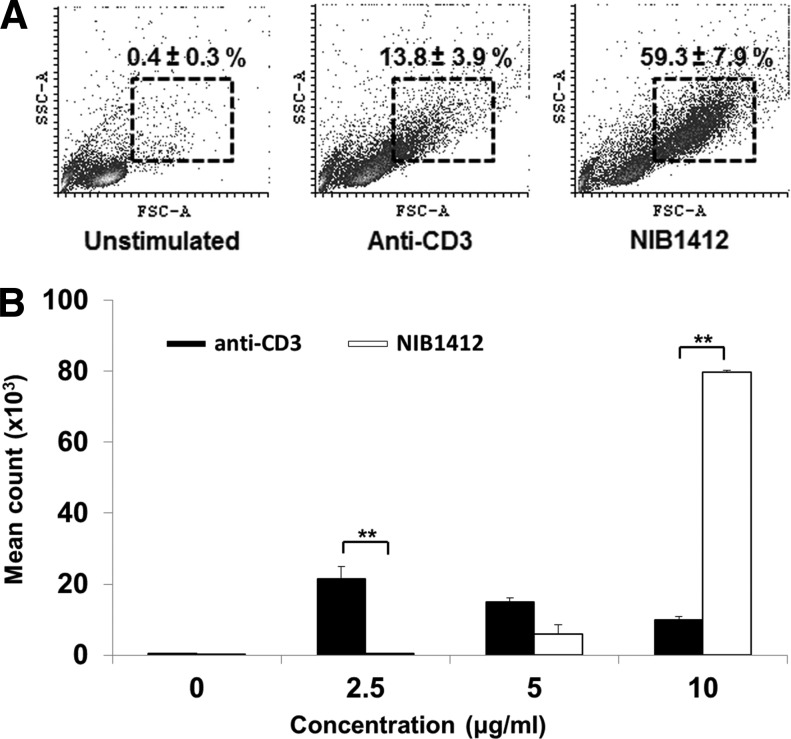
CD28SA-activated CD4^+^ T_EMs_ display a hyperactive phenotype. **(A)** Size (FSC) and granularity (SSC) were quantified by flow cytometry. FSC and SSC were measured on purified human CD4^+^ T_EMs_ stimulated with anti-CD3 or CD28SA for 48 hours. Percentages ± SD indicate T cell blasts. The data are representative of four independent experiments. **(B)** Human CD4^+^ T_EMs_ were stimulated with the indicated concentrations of plate-bound antibodies (anti-CD3 or NIB1412) and proliferation was measured 3 days postactivation by ^3^H-labeled thymidine incorporation. The vertical axis represents mean cpm ± SD from triplicate wells. The data are representative of four independent experiments (**p < 0.01; unpaired, two-tailed Student's *t*-test). NIB1412–CD28SA. CD28SA, CD28 superagonist; cpm, counts per minute; FSC, forward scatter; SD, standard deviation; SSC, side scatter; T_EMs_, effector memory T cells.

T cell activation triggers metabolic programs that promote biomass generation, which is evident by an increase in cell size, termed blasting. We used a TGN1412-like CD28SA mAb, termed NIB1412 to stimulate CD4^+^ T_EMs_ and demonstrate that the percentage of blasting T cells in the CD28SA-stimulated condition is about 4-fold (59% compared to 14% with anti-CD3) greater than in the CD3-activated condition (Fig. 1A). We also confirmed the hyperactive phenotype of CD28SA-activated T cells as evidenced by their enhanced proliferative response (Fig. 1B).

### CD28SA stimulation maximizes OXPHOS potential

**FIG. 2.**
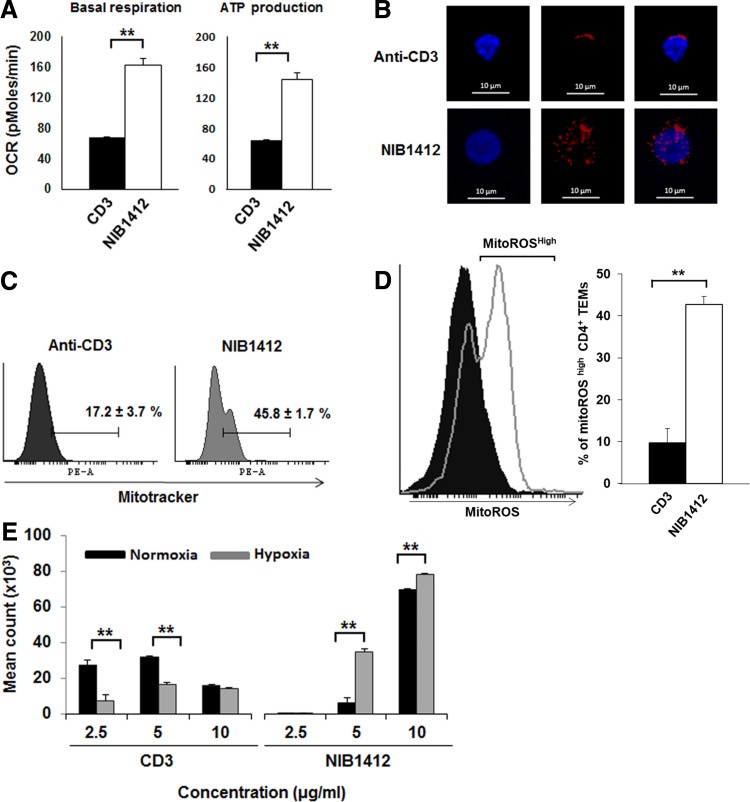
CD28SA stimulation maximizes OXPHOS potential. **(A)** OCR (pMoles/min) of preactivated human CD4^+^ T_EMs_ were measured using the seahorse XF96 extracellular flux analyzer in real time, under basal conditions and in response to sequential addition of Oligomycin (1 μM), 2,4-DNP (160 μM), and antimycin A and rotenone (1 μM). Mean OCR values representing basal respiration and ATP production are presented as bar charts. The bars represent the mean ± SD. The data are representative of three independent experiments (**p < 0.01; unpaired, two-tailed Student's *t*-test). **(B)** IF images of mitochondria. IF images show anti-CD3- and CD28SA-stimulated human CD4^+^ T_EMs_ stained with MitoTracker (red) and Hoechst (blue); far right quadrant represents merged image of the two stains; scale bars represent 10 μm. **(C)** Mitochondria were stained with MitoTracker Deep Red or **(D)** MitoSOX Red Mitochondrial Superoxide Indicator, and the staining intensities were quantified by flow cytometry. Data represent the mean ± SD percentage of cells staining positive for mitochondria, oitoROS, with the mean percentage of mitoROS present in activated CD4^+^ T_EMs_. Mean ± SD were obtained from three independent experiments (**p < 0.01; unpaired, two-tailed Student's *t*-test). **(E)** Human CD4^+^ T_EMs_ were stimulated with the indicated concentrations of plate-bound antibodies (anti-CD3 or NIB1412) and incubated in normoxic (20% O_2_) or hypoxic (5% O_2_) conditions. Proliferation was measured 3 days postactivation by ^3^H-labeled thymidine incorporation. The vertical axis represents mean cpm ± SD from triplicate wells. The data are representative of three independent experiments (**p < 0.01; unpaired two-tailed *t*-test). NIB1412–CD28SA. IF, immunofluorescent; mitoROS, mitochondrial reactive oxygen species; OCR, oxygen consumption rates; OXPHOS, oxidative phosphorylation.

To assess the contribution of OXPHOS to meet the energy requirements during hyperactivation of CD4^+^ T_EMs_ upon CD28SA stimulation, we used extracellular flux analysis to measure oxygen consumption rates (OCR), basal respiration, ATP production, maximal respiration and spare respiratory capacity (SRC). CD28SA-stimulated T cells had higher basal respiration and ATP production when compared with anti-CD3-activated CD4^+^ T_EMs_ (Fig. 2A). To determine whether CD28SA-stimulated T cells had more active mitochondrial mass, we stained CD4^+^ T_EMs_ with MitoTracker Deep Red and found that CD28SA stimulation led to a higher quantity of actively respiring mitochondria than when activated with anti-CD3 mAbs (Fig. 2B, C). CD28SA-stimulated T cells also produced more mitochondrial reactive oxygen species (mitoROS) than anti-CD3-activated CD4^+^ T_EMs_ (Fig. 2D), which is reflective of enhanced mitochondrial electron transport activity. Collectively, these results indicate that CD28SA-activated cells maximize their OXPHOS potential, which is supported by their greater active mitochondrial mass. To test whether CD28SA-stimulated cells are reliant on OXPHOS for their hyperproliferation, we cultured activated CD4^+^ T_EMs_ under hypoxic or normoxic conditions. While, CD3-stimulated cells proliferated more in normoxic than in hypoxic condition, CD28SA-stimulated cells proliferated in both the conditions with higher rate of proliferation in hypoxic condition (Fig. 2E). This suggests that CD28SA-activated T cells have a flexible metabolic program that reduces the reliance on OXPHOS for proliferation.

### CD28SA stimulation maximizes glucose utilization

**FIG. 3.**
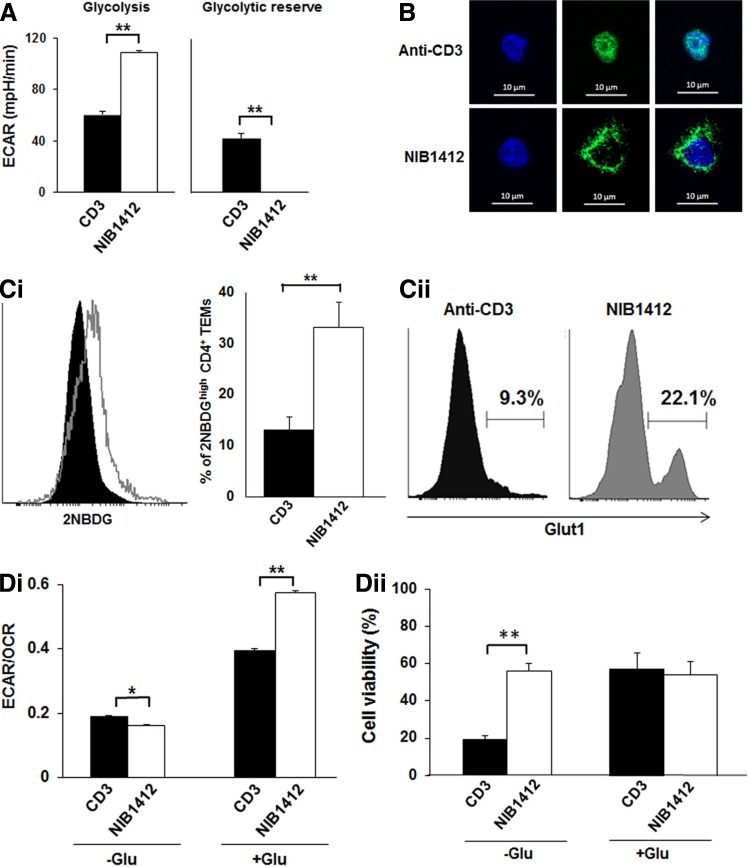
CD28SA stimulation maximizes glucose utilization. **(A)** ECAR (mpH/min) of preactivated human CD4^+^ T_EMs_ were measured using the seahorse XF96 extracellular flux analyzer in real time, under basal conditions, and in response to sequential addition of glucose (10 mM), Oligomycin (1 μM), and 2-DG (100 mM). Mean ECAR values representing glycolysis and glycolytic reserve are presented as bar charts. The bars represent the mean ± SD. The data are representative of three independent experiments (**p < 0.01; unpaired two-tailed *t*-test). **(B)** Immunofluorescence images show anti-CD3- and CD28SA-stimulated human CD4^+^ T_EMs_ at 48 hours postactivation stained with anti-GAPDH (green) and DAPI (blue); scale bars represent 10 μm. Far right quadrant represents merged image of the two stains. **(C)** Human CD4^+^ T_EMs_ were stimulated with anti-CD3 or CD28SA for 48 hours. Cells were then incubated with 2-NBDG for 30 mins and the amount of 2NBDG uptake **(i)** was measured by flow cytometry, with the mean percent of 2-NBDG-positive CD4^+^ T_EMs_ presented in a bar graph. The bars represent the mean ± SD from three different experiments (**p < 0.01; unpaired two-tailed *t*-test). Cells were also stained for Glut1 expression **(ii)**. The data are representative of three independent experiments. **(D)** Basal ECAR and OCR of preactivated human CD4^+^ T_EMs_ were measured using the seahorse XF96 extracellular flux analyzer in real time, with the **(i)** ECAR/OCR ratio presented as bar charts. The bars represent the mean ± SD. The data are representative of three independent experiments (*p < 0.05, **p < 0.01; two-tailed unpaired *t*-test). **(ii)** Cell viability of anti-CD3- and CD28SA-stimulated human CD4^+^ T_EMs_ cultured for 24 hours in the presence or absence of glucose. Percentage of viable cells was determined by using Trypan Blue. The data are representative of three independent experiments (**p < 0.01; unpaired two-tailed *t*-test). NIB1412–CD28SA. 2-DG, 2-deoxy-d-glucose; ECAR, extracellular acidification rate.

To assess glucose handling during CD28SA stimulation, we measured ECAR, a marker of glycolysis, and found that CD28SA-stimulated CD4^+^ T_EMs_ displayed significantly higher glycolysis and lacked glycolytic reserve compared with anti-CD3-stimulated cells (Fig. 3A). This result indicates that CD28SA-activated cells adopt a metabolic program that maximizes basal glycolytic rate. We tested whether the high glycolytic rate was associated with altered glycolytic machinery in CD4^+^ T_EMs_. The enzyme GAPDH is a critical component of the glycolytic pathway that generates pyruvate from glucose (Supplementary Fig. S1) with cytoplasmic distribution of GAPDH being indicative of glycolytic activity. We examined the cellular distribution of GAPDH by immunofluorescence and observed that the distribution of GAPDH staining was coincident with nuclear contours in anti-CD3-stimulated CD4^+^ T_EMs_ (Fig. 3B). In contrast, CD28SA-stimulated CD4^+^ T_EMs_ had more dispersed cytosolic distribution of GAPDH (Fig. 3B).

Increased glycolytic rates need to be fuelled by a commensurate increase in the uptake of glucose, which is predominantly mediated by the glucose transporter, GLUT1 in T cells. The uptake of the fluorescent glucose analog 2-NBDG (Fig. 3C-i) and cell surface expression of GLUT1 (Fig. 3C-ii) were both higher in CD28SA-stimulated CD4^+^ T_EMs_ than in CD3-stimulated cells. The ratio of the basal ECAR to OCR indicates the cellular preference for glycolysis versus OXPHOS metabolic programs.^(12)^ Figure 3D-i shows the ECAR/OCR ratio of CD28SA- and anti-CD3-stimulated T cells cultured (in base media with glutamine only) in the absence of glucose (−Glu) or in the presence of glucose (+Glu). In the −Glu condition the ECAR/OCR ratio was significantly lower in CD28SA-stimulated cells than anti-CD3-stimulated cells. In the +Glu condition CD28SA-stimulated cells had a significantly higher ECAR/OCR ratio than anti-CD3-stimulated cells. These plots demonstrate that in the presence of glucose, CD28SA-stimulated cells preferentially drive glycolysis, whereas anti-CD3-stimulated cells also drive OXPHOS. Collectively these results show that CD28SA stimulation drives an adaptable metabolic program that maximizes glucose utilization to drive both OXPHOS and glycolysis depending on glucose availability. This is further demonstrated by cell viability in glucose-deficient conditions wherein viability of CD28SA-stimulated cells was unaffected after 24 hours while more than 50% of the CD3-stimulated cells died (Fig. 3D-ii).

### CD28SA-activated T cells are metabolically programmed to favor lipogenesis

**FIG. 4.**
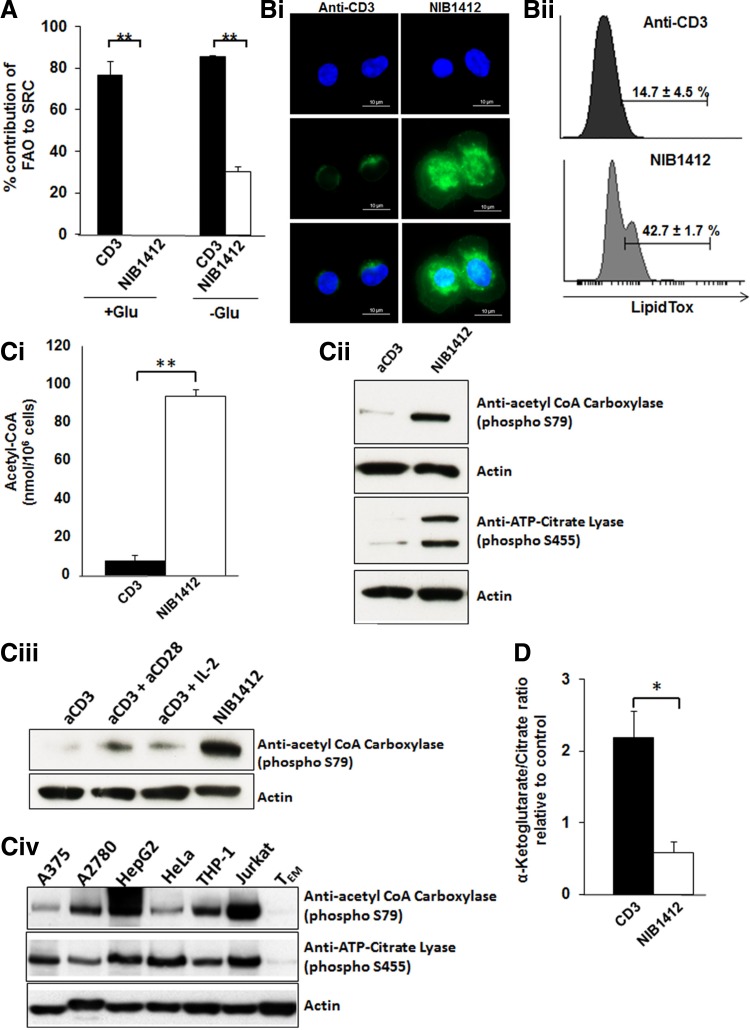
CD28SA activation induces *de novo* lipogenesis. **(A)** OCR (pMoles/min) of preactivated human CD4^+^ T_EMs_ were measured using the seahorse XF96 extracellular flux analyzer in real time, under basal conditions, and in response to sequential addition of Oligomycin (1 μM), 2,4-DNP (160 μM) and antimycin A and rotenone (1 μM). Etomoxir (200 μM) or medium was injected after 2,4-DNP injection. The SRC (quantitative difference between maximal uncontrolled OCR ± etomoxir treatment and initial basal OCR) was calculated and presented as the percentage contribution of FAO to SRC in bar charts. The bars represent mean ± SD. The data are representative of three independent experiments (**p < 0.01; unpaired two-tailed *t*-test). **(B)** Immunofluorescence images show **(i)** anti-CD3- and CD28SA-stimulated CD4^+^ T_EMs_ at 48 hours postactivation stained with LipidTOX (green) and DAPI (blue); scale bars represent 10 μm. Far bottom quadrant represents merged image of the two stains. **(ii)** LipidTOX staining was quantified by flow cytometry. Data represent the mean percent of cells ± SD (*n* = 3) staining positive for neutral lipids. **(C-i)** Relative levels of acetyl-CoA in anti-CD3- and NIB1412-stimulated T_EMs_ at 48 hours postactivation; mean ± SD of triplicates (**p < 0.01). **(ii)** Expression of p-ACC (250 kDa) and p-ACL (125 kDa) by western blot. **(iii)** Expression of p-ACC with IL-2 condition, in anti-CD3- and NIB1412-stimulated T_EMs_ at 48 hours postactivation, and **(iv)** Expression of p-ACC and p-ACL in human malignant melanoma cells (A375), human ovarian tumor cells (A2780), hepatocellular cells (HepG2), human cervical cancer cells (HeLa), human monocytic leukemia cells (THP-1), human T cell lymphoblast-like cells (Jurkat), and unstimulated T_EMs_ was determined by western blot. Actin (45 kDa) was used as a loading control. **(D)** Alpha-ketoglutarate-to-citrate ratio in anti-CD3- and CD28SA-stimulated human CD4^+^ T_EMs_ at 48 hours postactivation, presented as bar charts. The bars represent the mean ± SD of triplicate measurements (*p < 0.05; unpaired two-tailed Student's *t*-test). NIB1412–CD28SA. ACC, acetyl-CoA carboxylase; ACL, ATP-citrate lyase; FAO, fatty acid oxidation; SRC, spare respiratory capacity.

To determine whether mitochondrial fatty acid oxidation (FAO) contributes to OXPHOS, we measured changes in SRC in the presence of the Carnitine palmitoyl transferase (CPT1a) inhibitor, etomoxir (CPT1a is involved in transfer of fatty acids from cytosol to the mitochondria). In both +Glu and −Glu conditions, around 80% of FAO contributed to the SRC of CD3-stimulated CD4^+^ T_EMs_. In contrast, only about 30% of FAO contributed to the SRC of CD28SA-stimulated cells in −Glu while there was no contribution of FAO to the SRC in the +Glu conditions (Fig. 4A). The low rate of FAO in CD28SA-activated CD4^+^ T_EMs_ suggested the possibility that these cells are potentially increasing their lipid reserve. LipidTox staining revealed that CD28SA stimulation induces a significantly greater accumulation of neutral lipids in CD4^+^ T_EMs_ than when activated with anti-CD3 mAbs (Fig. 4B-i). This was confirmed and quantified by flow cytometry (Fig. 4B-ii). We next measured the acetyl-CoA content and demonstrate that total acetyl-CoA levels were significantly higher in CD28SA-stimulated CD4^+^ T_EMs_ than in CD3-stimulated cells (Fig. 4C-i). The increased lipogenesis in CD28SA-stimulated cells is reflected by the hyperactivity of the lipogenic enzymes, ACL and ACC, as determined by their phosphorylation status (Fig. 4C-ii). Hyperactivation of ACL and ACC has been reported in tumor cells and we confirmed this in several types of tumor cell lines (Fig. 4C-iv).

To rule out the influence of the CD28SA-secreted IL-2 on the *de novo* lipogenesis, we also activated CD4^+^ T_EMs_ with anti-CD3 in combination with IL-2 (aCD3+IL-2), and with anti-CD3 in combination with conventional anti-CD28 (aCD3+aCD28). As can be seen in the results, CD28SA-stimulated cells had significantly greater ACC phosphorylation than any of the other conditions (Fig. 4C-iii). This indicates that the enhanced lipogenic program in CD28SA-activated CD4^+^ T_EMs_ is not secondary to IL-2 effects. We observed that CD3-stimulated CD4^+^ T_EMs_ possessed a high relative α-KG/citrate ratio of 2.2 ± 0.3, suggesting ongoing reductive glutamine metabolism (Fig. 4D). In contrast, CD28SA-stimulated cells had a low relative α-KG/citrate ratio of 0.6 ± 0.1 indicative of an absence of reductive glutamine carboxylation (Fig. 4D).

Collectively, these results show that CD28SA-stimulated cells have a dominant program of endogenous biosynthesis of fatty acids, which was not accompanied by preferential reductive glutamine metabolism. Our results show that CD28SA stimulation induces a tumor cell-like metabolic phenotype, where increased glycolytic flux correlates with elevated *de novo* lipogenesis and reduced FAO.

## Discussion

The metabolic programming of T cells is an area of intense investigation with implications for therapeutic intervention in many disease areas.^(13–16)^ We and others have previously shown that CD28SA activation results in a hyperactive T cell phenotype.^(4,17)^ Activated immune cells prefer glycolysis over OXPHOS as it is around two orders of magnitude faster for biomass accumulation and proliferation.^(18)^ Nevertheless, OXPHOS is necessary for cell-surface expression of the IL-2 receptor and thus vital for lymphocyte proliferation.^(19)^

In the current study, we find that CD28SA-stimulated T_EMs_ cells had higher levels of ATP production, basal respiration, and actively respiring mitochondria compared with anti-CD3-activated T_EMs_. The high oxygen consumption and ATP production is indicative of enhanced rates of OXPHOS, which occurs primarily in the mitochondria.^(20)^ Studies have shown that an increase in mitochondrial mass results in an increase in overall mitochondrial function, with a boost in residual OXPHOS capacity and an increase in overall ATP-generating capacity.^(21,22)^ Furthermore, our observation of increased mitoROS in CD28SA-stimulated T_EMs_ cells is reflective of enhanced mitochondrial electron transport activity.^(23)^ MitoROS production is regulated by mitochondrial membrane potential (Δψm), metabolic state of mitochondria, and oxygen concentration.^(24)^ Mitochondrial mass and Δψm are known to be significantly increased in cells during cell cycle, particularly when they are in S phase^(25)^ and we have shown in a previous study^(4)^ that a high proportion of CD28SA-stimulated cells are in S-phase at 48 hours postactivation and onward. Thus, the elevated mitochondrial mass and mitoROS might be due to the rapid cell proliferation induced by CD28SA activation. We also observed that CD28SA-stimulated cells proliferated in both normoxic and hypoxic conditions with higher rate of proliferation in the latter. These observations indicate that CD28SA-activated T cells have a flexible metabolic program that reduces the reliance on OXPHOS for proliferation.

We observed that CD28SA stimulation maximizes glucose utilization with higher glycolysis and a deficient glycolytic reserve. The T cell machinery has to be configured to support the higher glycolytic rate. Studies have shown that GAPDH shuttles between nuclear and cytoplasmic compartments, with cytoplasmic GAPDH being active in glycolysis,^(26)^ while nuclear GAPDH is thought to be involved in the initiation of apoptotic cascades^(27)^ and promoting gene transcription.^(28,29)^ We observed a predominant cytosolic distribution of GAPDH in CD28SA-stimulated T_EMs_ as well as increased cell surface GLUT1 expression, supportive of enhanced glucose uptake and utilization.^(30)^ In keeping with a role for CD28 in this process, CD28 costimulation (in addition to TCR activation), acting through the PI3K/Akt pathway is an important component in increasing glycolytic flux essential for T cell proliferation.^(31)^ Interestingly, the adaptable metabolic program induced by CD28SA in T cells (which is accompanied by supportive cellular changes) is reminiscent of the metabolic and cellular changes seen in tumor cells. For example, a variety of tumor cells show upregulation of *GAPDH* together with its accumulation in the cytoplasm,^(29)^ exhibit elevated levels of glucose uptake and glucose transporters, and^(32)^ can survive *in vitro* in the complete absence of glucose if supplied with glutamine.^(33)^

Mitochondrial FAO is a source of acetyl CoA that is used in the TCA cycle for energy generation through OXPHOS.^(34)^ CPT1a is a metabolic enzyme that controls the rate-limiting step in mitochondrial FAO.^(35)^ IL-2-secreting CD8^+^ effector memory T cells store exogenous long-chain fatty acids in lipid droplets, which are then mobilized for mitochondrial FAO.^(8)^ In contrast, CD8^+^ memory T cells were shown to use extracellular glucose to synthesize fatty acids for neutral lipid stores,^(8)^ which then undergo lipolysis to supply fatty acids for FAO as well as for incorporation into cellular membrane structures.

The lipid reserve of T cells is determined by the balance between lipogenesis and rate of FAO.^(8)^ *De novo* fatty acid biosynthesis is not only required to synthesize new membranes for cell proliferation but also to facilitate the formation of lipid rafts for increased signaling of cell surface receptors.^(36)^ Accumulated neutral lipid stores are then mobilized to provide free fatty acids to promote production of signaling lipids, for incorporation into cellular membranes or for the production of ATP through β-oxidation.^(37)^ Although the dynamics of fatty acid utilization for FAO has not been shown in CD4^+^ T_EMs_, in this report we propose that CD28SA stimulation enables CD4^+^ T_EMs_ to promote endogenous biosynthesis of fatty acids similar to cancer cells, which are known to undergo a metabolic shift toward pronounced increase in *de novo* lipid biosynthesis.^(38)^

Lipogenesis requires the availability of acetyl-CoA, which is an essential substrate for the endogenous biosynthesis of fatty acids. Increased glycolysis can shunt glucose-derived pyruvate into the mitochondria to be decarboxylated to acetyl-CoA, and then condensed with oxaloacetate to form citrate.^(39)^ Citrate is then exported from the mitochondria through the malate citrate shuttle system and used as a substrate for ACL.^(40)^ ACL is a key cytosolic enzyme that catalyses the generation of acetyl-coenzyme A (CoA) from mitochondria-derived citrate. Acetyl-CoA is then carboxylated to malonyl-CoA by ACC, which subsequently results in fatty acid synthesis. The activity of ACL and ACC are increased by serine phosphorylation.^(41,42)^ We report that total acetyl-CoA levels were significantly higher along with increased activity of ACL and ACC in CD28SA-stimulated T_EMs_ than in CD3-stimulated cells. We also show that IL-2 derived from CD28SA-stimulated T cells^(43)^ did not contribute to the enhanced lipogenic program observed in CD28SA-activated T_EMs_. The rapid proliferation of several types of cancer cells is dependent on the enhanced activity of ACL, with elevated acetyl-coA levels and increased activity of lipid-metabolizing enzymes being increasingly recognized as a feature of cancer cells.^(44,45)^ This metabolic program is now observed in CD28SA-stimulated T_EMs_.

ACL and ACC represent committed steps in channeling glucose-derived metabolites toward a lipid biosynthetic fate, which is regulated by PI3K/Akt signaling.^(46)^ This is consistent with the activation of the PI3K pathway by engagement of CD28 by the superagonistic mAb.^(47)^ The acetyl CoA that feeds lipogenesis is primarily derived from citrate.^(48)^ The citrate can be produced from glucose-derived pyruvate that enters the TCA cycle. A recent study has shown that in cancer cells, glutamine can enter the TCA cycle and also become a source of citrate for *de novo* lipogenesis through reductive carboxylation of glutamine-derived α-KG^(49)^ (Supplementary Fig. S1). An elevated α-KG/citrate ratio was shown to be the principal driving force for reductive glutamine metabolism^(49)^ and we observed a low relative α-KG/citrate ratio in CD28SA-stimulated T_EMs_ cells indicative of an absence of reductive carboxylation, unlike anti-CD3-stimulated T_EMs_. Elevated glycolytic flux is associated with *de novo* lipogenesis and reduced FAO. CD28SA-stimulated T_EMs_ exhibited increased glycolytic flux similar to cancer cells where intermediate metabolites are channeled toward acetyl-CoA generation for use in *de novo* fatty acid biosynthesis.^(36)^

The tumor microenvironment (TME) is rendered hostile to the survival and function of tumor infiltrating effector T cells by the remodeling effects of tumor cells. Tumor cells achieve this through a number of mechanisms, including establishing a hypoxic milieu, depleting extracellular glucose, secreting metabolites that impair immunity and by enhancing expression of ligands for immune checkpoints such as PD-1 to maintain T cell exhaustion.^(50)^ Current immunotherapies based on checkpoint inhibitors target limited pathway/s involved in immune evasion. A comprehensive immunotherapeutic strategy should be aimed at increasing T cell fitness through countering multiple aspects of tumor cell remodeling of TME. Our results suggest that T cells that have been metabolically reprogrammed through the CD28SA pathway will have superior fitness in the TME owing to their increased tolerance to hypoxia and a flexible metabolic program, much alike tumor cells. Furthermore, we have also previously shown that CD28SA-activated T cells can escape inhibition through the PD-1 pathway.^(4)^ Our findings indicate that immunotherapies that induce T cell metabolic reprogramming similar to CD28SA-activated T cells will enhance the efficacy of tumor-infiltrating T cells. For example, it can be envisaged that chimeric antigen receptor T cells that are modified to exploit the metabolic reprogramming of the CD28SA pathway will have superior efficacy and persistence in the TME.

In summary, we have shown that CD28SA activation induces a tumor cell-like T cell metabolic program that is geared toward maximal glucose utilization, endogenous lipid synthesis, and less dependence on OXPHOS. Tumor cells frequently exhibit similar alterations in metabolic activity to support the increased production of metabolic intermediates for the synthesis of proteins, nucleic acids, and lipids. We have also defined ACL and ACC as the likely molecular components that support operation of this metabolic program. Our study provides insights into the remarkable capacity of T cells to adopt a variety of distinct metabolic programming depending on the pathways that are engaged.

Our results may also have implications and utility in drug development, as dysregulated metabolic patterns in immune cells treated with therapeutic mAbs could serve as novel biomarkers of hazard identification. For example, aberrant nutrient uptake and utilization, together with increased *de novo* lipid biosynthesis could be indicative of excessive immunostimulatory potential of the therapeutic mAb. Metabolic studies that compare a number of immunostimulatory therapeutic mAbs are required to examine this further.

## Supplementary Material

Supplemental data
