# Supplementary material for: CD28 Superagonistic Activation of T Cells Induces a Tumor Cell-Like Metabolic Program
Source: Monoclon Antib Immunodiagn Immunother. 2019 Apr 22;38(2):60–9. doi: 10.1089/mab.2018.0042 (PMC6634261; doi:10.1089/mab.2018.0042)
Supplement: Supplemental data [file Supp_Fig1.pdf]

Supplementary Data

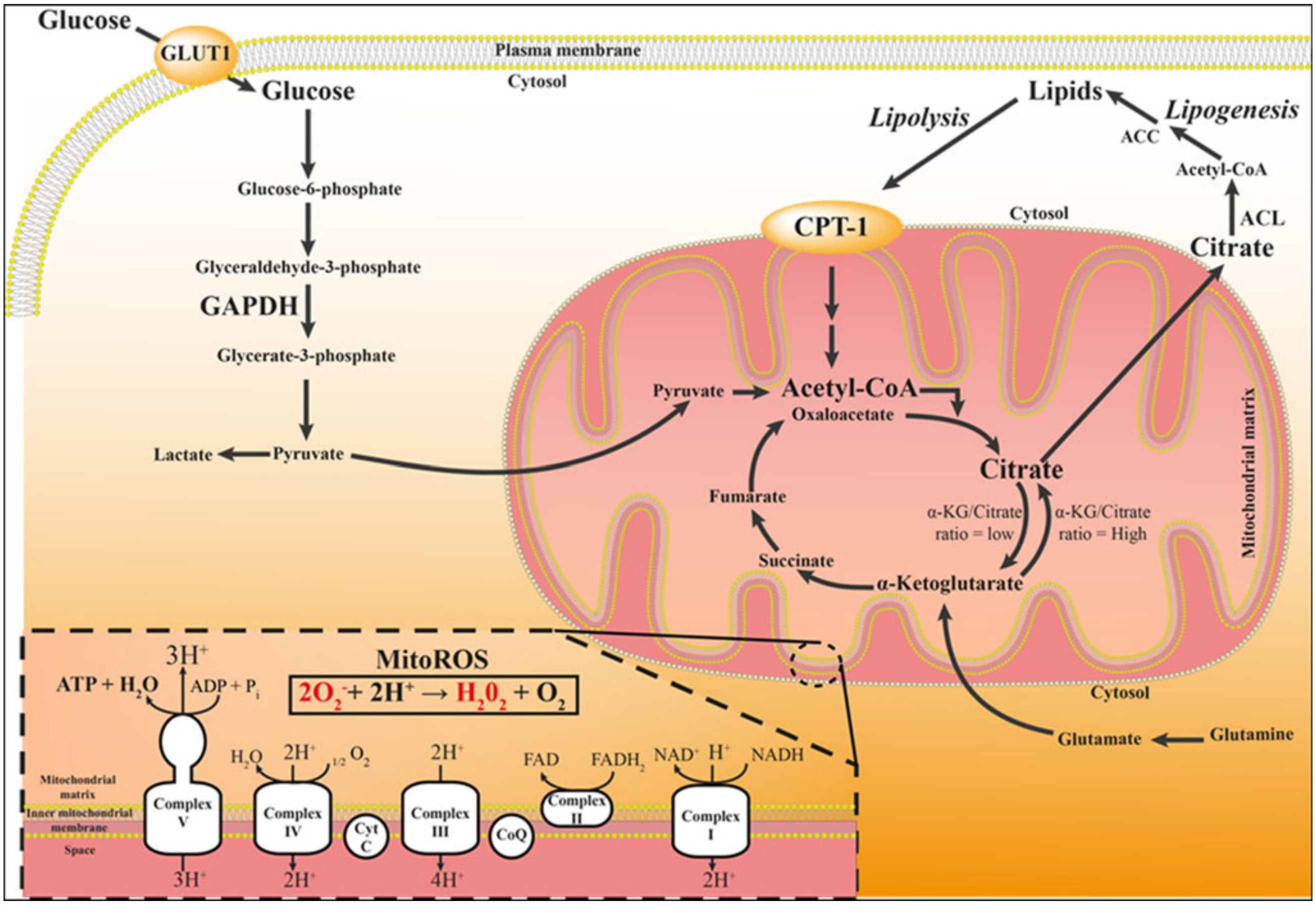

SUPPLEMENTARY FIG. S1. Illustrated diagram of glucose utilization and lipogenesis. A schematic illustration of glycolysis and *de novo* lipogenesis pathway is shown. Glucose is transported into the cell through Glut1. Pyruvate is the end product of glycolysis and is used either to produce lactate or is shuttled into the mitochondria. In the mitochondria, pyruvate is either oxidized to acetyl-CoA or converted to OAA (anaplerosis). acetyl-CoA and OAA together give rise to citrate, which enters the Krebs cycle. Citrate either continues with the next step in the Krebs cycle or leaves the mitochondria and is used by ACL in the cytosol to produce acetyl-CoA and OAA. In the cytosol, acetyl-CoA is carboxylated to malonyl-CoA ACC, which subsequently results in lipid synthesis. Fatty acids can become available from the lipolysis of lipid droplets, converted to acyl-carnitine for transport into the mitochondria through the carnitine shuttle and used as fuel for β-oxidation. The Krebs cycle also generates NADH and $FADH_2$, which donate electrons to the mitochondrial electron transport chain, which produces ROS. ACC, acetyl-CoA carboxylase; ACL, ATP-citrate lyase; CPT1, carnitine palmitoyltransferase I; GAPDH, glyceraldehyde 3-phosphate dehydrogenase; GLUT1, glucose transporter 1; mitoROS, mitochondrial reactive oxygen species; OAA, oxaloacetate.
